# *Uncaria tomentosa* Leaves Decoction Modulates Differently ROS Production in Cancer and Normal Cells, and Effects Cisplatin Cytotoxicity

**DOI:** 10.3390/molecules22040620

**Published:** 2017-04-12

**Authors:** Anita Kośmider, Edyta Czepielewska, Mieczysław Kuraś, Krzysztof Gulewicz, Wioleta Pietrzak, Renata Nowak, Grażyna Nowicka

**Affiliations:** 1 Department of Biochemistry and Pharmacogenomics, Faculty of Pharmacy with the Laboratory Medicine Division, and Center for Preclinical Studies, Medical University of Warsaw, 02097 Warsaw, Poland; grazyna.nowicka@wum.edu.pl; 2 Department of Clinical Pharmacy and Pharmaceutical Care, Faculty of Pharmacy with the Laboratory Medicine Division, Medical University of Warsaw, 02097 Warsaw, Poland; edyta.jaszewska@wum.edu.pl; 3 Department of Molecular Plant Physiology, Faculty of Biology, University of Warsaw, 02096 Warsaw, Poland; kuras@biol.uw.edu.pl; 4 Institute of Bioorganic Chemistry Polish Academy of Science, Poznań, 61704 Poznań, Poland; krysgul@ibch.poznan.pl; 5 Chair and Department of Pharmaceutical Botany, Medical University of Lublin, 20093 Lublin, Poland; wioletapietrzak@umlub.pl (W.P.); renata.nowak@umlub.pl (R.N.)

**Keywords:** *Uncaria tomentosa* decoction, tannin and alkaloids, HepG2 cells, ROS production, NF-κB activity, CDDP cytotoxicity

## Abstract

*Uncaria tomentosa* is a woody vine with a long history of use in traditional Peruvian medicine and nowadays supplements containing this vine as ingredient are available. Immunomodulating, anti-inflammatory and anticancer properties of *Uncaria tomentosa* have been suggested and attributed mainly to the presence of tetracyclic or pentacyclic oxindole alkaloids. However, the synergic action of different compounds occurring in extracts and modulation of redox processes may significantly influence the anticancer activity of *Uncaria tomentosa*. The aim of the present study was to investigate for the first time the cytotoxic effects of the tetracyclic alkaloids free aqueous extract (decoction) of dried *Uncaria tomentosa* leaf blades in normal and cancer cells, and to assess the effect of the tested extract on cisplatin (CDDP) cytotoxicity. Tested *Uncaria tomentosa* extract was not cytotoxic for NHDF cells, but demonstrated cytotoxic effect against HepG2 cells. The extract increased ROS production in HepG2 cells, which resulted in decreased GSH level, leading to apoptosis of these cells through activation of caspase-3 and caspase-7. A reduction of NF-κB active form was observed in cancer cells. In normal cells the extract did not affect ROS production, GSH level and NF-κB activity, and maintained cell viability. HepG2 cells incubation with *Uncaria tomentosa* decoction and simultaneously with CDDP resulted in an increase in CDPP cytotoxic activity against HepG2, while under the same conditions *Uncaria tomentosa* prevents NHDF cell viability reduction due to CDDP. The results indicate that *Uncaria tomentosa* leaves decoction modulates differently cancer and normal cells oxidative metabolism and, enhanced cytotoxicity of CDDP against cancer cells and at the same time increased normal healthy cells resistance to cisplatin. Further studies are needed to confirm our observations and to describe underlying molecular mechanism, and the potential usefulness of *Uncaria tomentosa* decoction in adjuvant therapy for cancer.

## 1. Introduction

Previous studies have revealed that different organic and aqueous extracts of *Uncaria tomentosa*, a Peruvian plant of the Rubiaceae family, contain biologically active substances, including immunostimulating pentacyclic oxindole alkaloids [1], ursane type pentacyclic triterpenes and quinovic acid glycosides, which were reported to possess anti-anti-inflammatory properties [2] and inhibit viral replication [3]. These extracts contained also carboxyl alkyl esters, including quinic acid derivatives that, according to the available data, reduced proliferation of neoplastic cells and stimulated DNA repair [4], as well as antioxidants as catechin monomers and proanthocyanidins [5]. Nowadays dietary supplements containing this vine as ingredient are available and used in anti-cancer therapy.

In vitro studies have demonstrated antiproliferative and proapoptotic effects of *Uncaria tomentosa* bark extracts on various cell lines, including human leukemia cells K562 and HL60, EBV-transformed B lymphoma cells [6] and breast cancer cells MCF7 [7]. Ethyl acetate extract of *Uncaria tomentosa* in HL60 cells caused changes in mitochondrial membrane potential, release of cytochrome c to the cytosol and caspase-3 activation [8].

Although, in in vitro studies, organic extracts containing large amounts of oxindole alkaloids [9,10] and pure alkaloids isolated from *Uncaria tomentosa* [11,12] inhibited proliferation of neoplastic cells, in vivo in mice bearing Lewis lung carcinoma [10], a stronger inhibitory effect on cancer development was caused by water extracts with low alkaloid content. Water extracts contain polar compounds, which are more available to the body. It has been also suggested that the anti-inflammatory and probably anticancer properties of *Uncaria tomentosa* may be related to a synergic action of different compounds [13] and modulation of redox processes may play a pivotal role in the anticancer activity of this plant [14].

The aim of the present study was to investigate for the first time the cytotoxic activity of tetracyclic alkaloid free and tannins rich aqueous extract (decoction) of dried *Uncaria tomentosa* leaf blades, and its effect on ROS production in human hepatoma, HepG2 cells and normal human dermal fibroblast, NHDF. We assessed also the influence of this extract on cisplatin cytotoxicity against cancer cells and in normal healthy cells to give an insight in potential usefulness of *Uncaria tomentosa* in adjuvant treatment for cancer. Previous reports focused on the effects of bark and root extracts of *Uncaria tomentosa* on cancer cells, while leaves contain similar active compounds and appropriate material can be collected without significant plant damage [15]. The extract used in this study was prepared according to commonly accepted procedure for decoction preparation of *Uncaria tomentosa* by heating dried plant leaves in water.

## 2. Results and Discussion

### 2.1. Composition of Studied Decoction from Uncaria tomentosa Leaves

Plant extracts are extensively tested for anticancer activity, which may be a result of their antioxidant properties and apoptosis induction capacities, and in a future such extracts could be useful as an adjuvant treatment for cancer [2,16]

**Figure 1:**
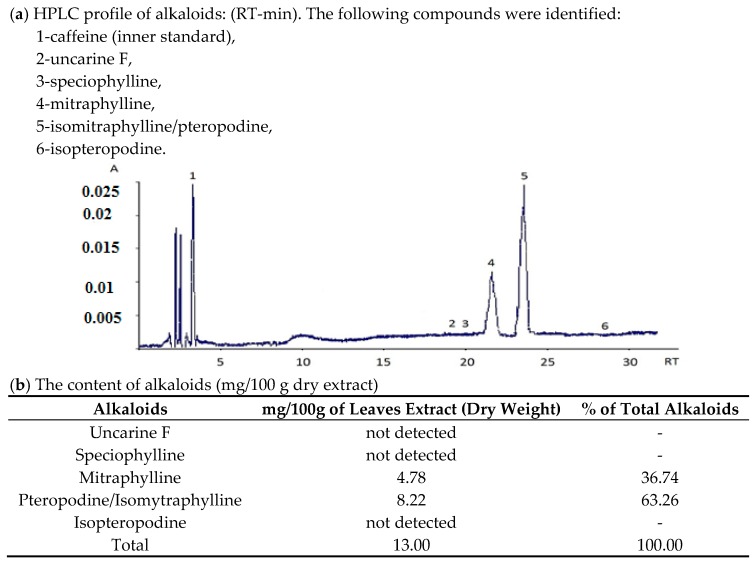
HPLC analysis of alkaloids in aqueous extract from leaves of *Uncaria tomentosa*.

The decoction (water extract) from *Uncaria tomentosa* leaves contains pentacyclic oxindole alkaloids (13% of dry extract mass) and is free of teracyclic alkaloids (Figure 1). Pentacyclic oxindole alkaloids: mitraphylline and pteropodine found in the extract were reported to inhibit the proliferation of cancer cells and exhibit immunomodulating properties [17,18]. It was recognized that tetracyclic alkaloids can significantly reduce activity of pentacyclic alkaloids [19], and according to the U.S. Pharmacopeia only extracts free of tetracyclic oxindole alkaloids may be used in humans for research and/or therapeutic purposes [20]. The tested extract contains also phenolic compounds (Table 1), including condensed tannins which belong to proanthocyanidins and possess antioxidant activity [2,21].

### 2.2. Effect of Uncaria tomentosa Decoction on Cancer and Normal Cell Viability

**Figure 2:**
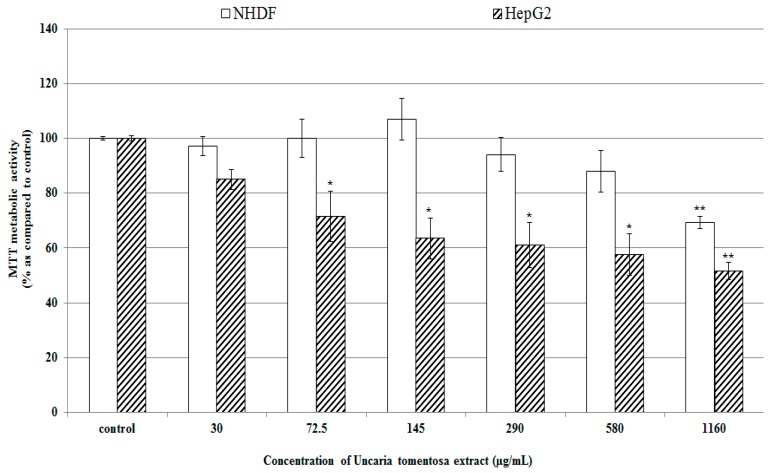
Cell viability assessed by MTT mitochondrial conversion. NHDF and HepG2 cells were treated with *Uncaria tomentosa* leaves extract (30–1160 µg/mL) for 72 h at 37 °C. Then, the mitochondrial metabolic activity of cells was measured by MTT assay. Data are expressed as means ± SD from three independent experiments performed in triplicate. Statistically significant differences: * *p* < 0.05 and ** *p* < 0.001 refer to the control (untreated cells).

The extract from *Uncaria tomentosa* leaves was recognized to be not toxic for NHDF cells, while significantly decreased cell viability was observed only when high extract concentrations >1 g/mL were used. In the present study treatment of HepG2 cells with this extract caused a significant concentration dependent decrease in cell viability (Figure 2), and based on the results of MTT assay IC_50_ (50% viability inhibition concentration) of 580 µg/mL was determined.

**Figure 3:**
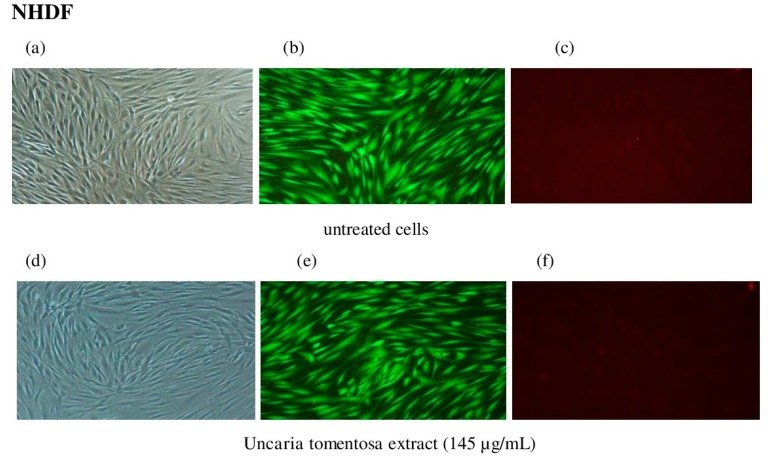

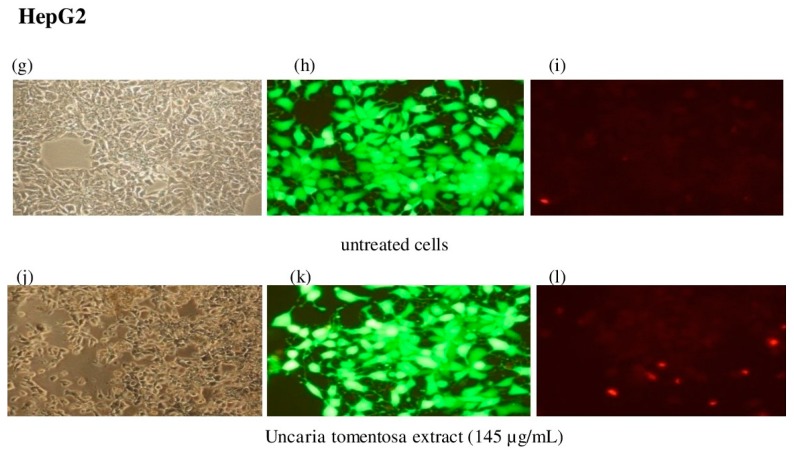
Morphologic changes of NHDF (**a**–**f**) and HepG2 (**g**–**l**) cells after treatment with *Uncaria tomentosa* extract. Untreated cells (**a**,**g**) observed in the light microscope (100×); (**b**,**h**) in fluorescence (200×) after calcein-AM staining, (**c**,**i**) in fluorescence (200×) after PI staining. Cells treated with 145 µg/mL of the extract observed in fluorescence after calcein-AM (**e**,**k**) and PI staining (**f**,**l**), respectively. The calcein in viable cells emits green fluorescence, while PI emits red fluorescence only in dead cells.

Cells incubated with the extract and control cells were also observed in fluorescence (Figure 3). With increasing concentration of the tested extract there was a progressive decrease in the number of viable HepG2 cells stained green with calcein, and an increase in the number of dead cells stained red with propidium iodide (PI) [22]. At the extract concentration of 145 µg/mL (Figure 3j–l) 22% of HepG2 cells were found stained red and this number was significantly higher than in control cells (* *p* < 0.05). In fluorescence images in NHDF cells incubated under similar conditions as HepG2 cells no significant changes in cell morphology and in quantity of red stained cells were noted (Figure 3a–f).

### 2.3. Effect of Uncaria tomentosa Decoction on Cell Apoptosis

**Figure 4:**
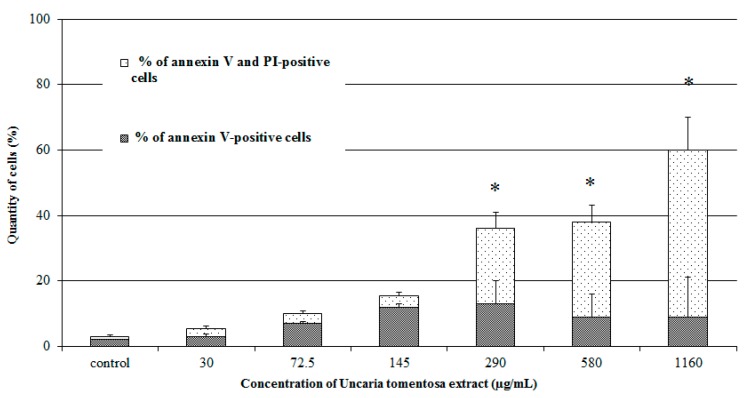

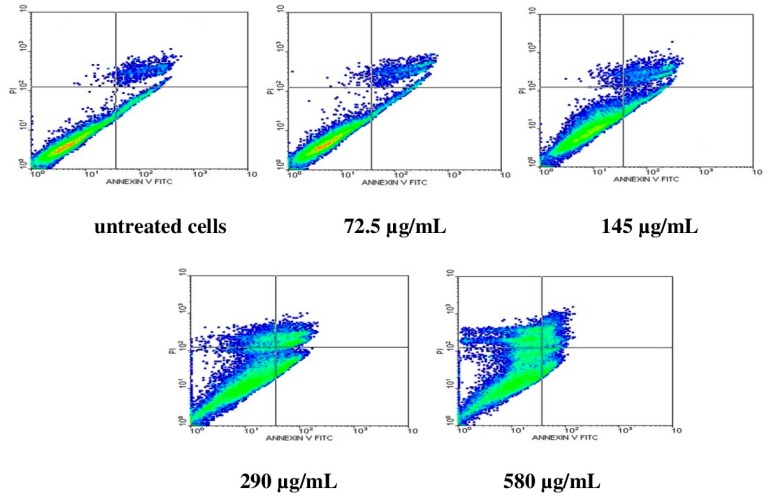
The number of HepG2 cells in early (An^+^/PI^−^) and late (An^+^/PI^+^) apoptosis evaluated using annexin V-FITC/PI staining followed by flow cytometric analysis. Cells were treated with *Uncaria tomentosa* leaves extract for 72 h. Data are expressed as means ± SD from two independent experiments performed in triplicate. Statistically significant difference * *p* < 0.05 refers to the control (untreated) cells. Representative histograms of control cells and cells treated with the extract (72.5–580 µg/mL) are shown.

Incubation of HepG2 cells with the *Uncaria tomentosa* leaves decoction at the concentrations of 72.5 µg/mL and −145 µg/mL resulted in an increase in the number of Annexin positive cells corresponding to apoptotic cells, while not significant differences in the number of Annexin positive and PI positive cells were observed. *Uncaria tomentosa* decoction concentrations higher than 145 µg/mL caused a significant increase (* *p* < 0.05) in the number of late apoptotic (Annexin positive and PI positive) or also necrotic cells (Figure 4).

Under similar conditions in NHDF cells no effect of *Uncaria tomentosa* decoction at the concentrations of 72.5 µg/mL and 145 µg/mL on the number of Annexin-positive as well as Annexin-positive and PI positive cells was found (data not shown).

### 2.4. Effect of Uncaria tomentosa Decoction on ROS Production and GSH Level

The changes in HepG2 cells viability might be associated with functional changes in the mitochondrial electron transport chain and development of oxidative stress, therefore, the production of ROS in HepG2 and NHDF cells was determined.

**Figure 5:**
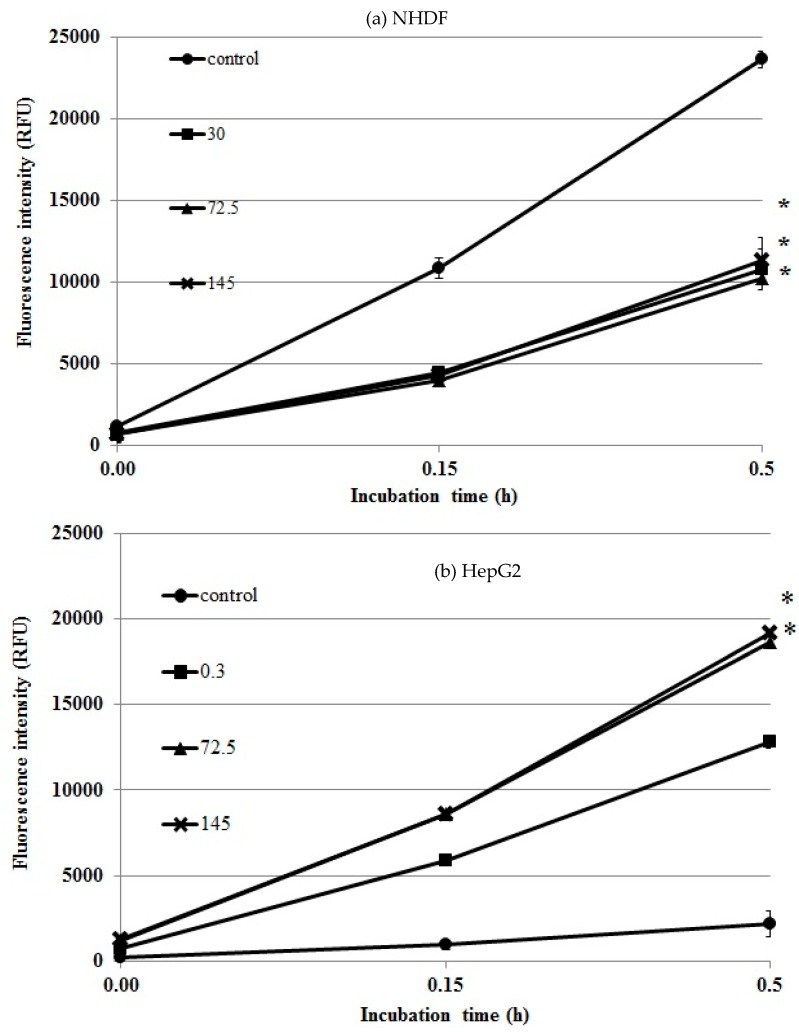
ROS formation in (**a**) NHDF and (**b**) HepG2 cells treated with *Uncaria tomentosa* leaves extract. Cells were exposed to extract (30–145 µg/mL) for 1 h at 37 °C and then were treated with 250 µM DCFH-DA. Control represents untreated cells. Results are presented as means of three independent experiments performed in triplicate. Level of significance was * *p* < 0.05 as compared to the control (untreated cells).

In HepG2 cells the control plot (for cells without the extract) as shown in (Figure 5b) lies below the plots of DCF intensity in cells incubated with different *Uncaria tomentosa* concentrations. This indicates an increase in cellular ROS production caused by the *Uncaria tomentosa* extract. The highest rate of ROS release was observed during the initial 0.5 h of incubation, while after 1 h, ROS levels became stabilized. *Uncaria tomentosa* extract, increased ROS formation, and a statistically significant increase (* *p* < 0.05) was seen. Under similar experimental conditions in NHDF (Figure 5a) cells was observed opposite effect of our *Uncaria tomentosa* extract on ROS production. All tested doses resulted in significantly (* *p* < 0.05) lower levels of fluorescence intensity as compared to controls indicating lower ROS production by *Uncaria tomentosa* treated NHDF cells than by control (untreated) NHDF cells.

**Figure 6:**
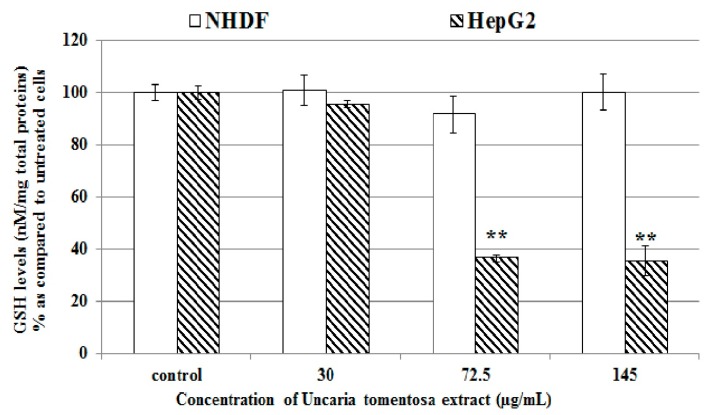
Influence of our *Uncaria tomentosa* leaves extract on GSH levels in HepG2 and NHDF cells. Cells were treated with tested extract (30–145 µg/mL) for 4 h at 37 °C and intracellular concentration of GSH were measured as described in the Materials and Methods section. Data are expressed as means ± SD from two independent experiments performed in triplicate. Statistically significant difference ** *p* < 0.001 refers to the control (untreated cells).

Our data indicate that tested *Uncaria tomentosa* extract may induce cytotoxic effect in cancer cells by stimulation of ROS overproduction. Glutathione (GSH) plays a significant role in maintaining oxidative-reductive balance in cells, and its level is an indicator of response to oxidative stress. We have shown that in HepG2 cells the *Uncaria tomentosa* extract caused a reduction of GSH levels and significant (** *p* < 0.001) GSH decrease was observed when *Uncaria tomentosa* concentration of 145 µg/mL was used (Figure 6).

*Uncaria tomentosa* extract in NHDF cells (Figure 6) did not cause significant changes in GSH levels as compared to control cells. Decrease in GSH levels predisposes cells to enter the pathway of death, while the persistently increased GSH levels in cancer cells can be responsible for their resistance to chemotherapeutic agents [23].

### 2.5. Effect of Uncaria tomentosa Decoction on Caspase-3 and Caspase-7 Activity

**Figure 7:**
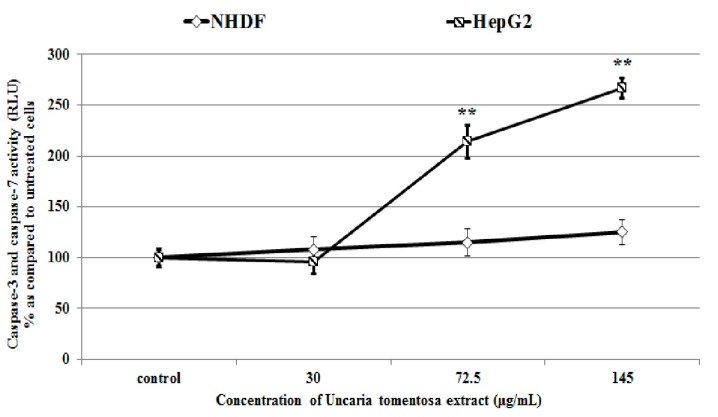
Changes in caspase-3 and caspase-7 activity in HepG2 and NHDF cells after 4 h at 37 °C incubation with *Uncaria tomentosa* leaves extract (30–145 µg/mL) according to the manufacturer’s protocol. Data are presented as means ± SD from two independent experiments performed in triplicate. Statistically significant differences: ** *p* < 0.001 refer to the control (untreated cells).

Modulation of the redox processes by tested extract may play a pivotal role in the loss of cell viability. ROS overproduction and decreased GSH levels may lead to destruction of intracellular structures and the cells may enter the pathway of death as indicated by observed changes in cell morphology and in the number of Annexin positive cells. Apoptotic effect of *Uncaria tomentosa* in HepG2 cells was confirmed by the activation of effector caspase-3 and caspase-7. In HepG2 cells the tested extract at the concentrations ≤145 µg/mL increased effector caspase-3 and caspase-7 activity by 250% (** *p* < 0.001, Figure 7) as compared to the basal activity (untreated cells). In NHDF cells the effect of the extract on these caspases was not significant as compared to untreated cells-control (Figure 7). These results are in agreement with the recent findings that *Uncaria tomentosa* induced cancer cell death through caspase-3 dependent apoptosis [14].

### 2.6. Effect of Uncaria tomentosa Decoction on NF-κB Activity

**Figure 8:**
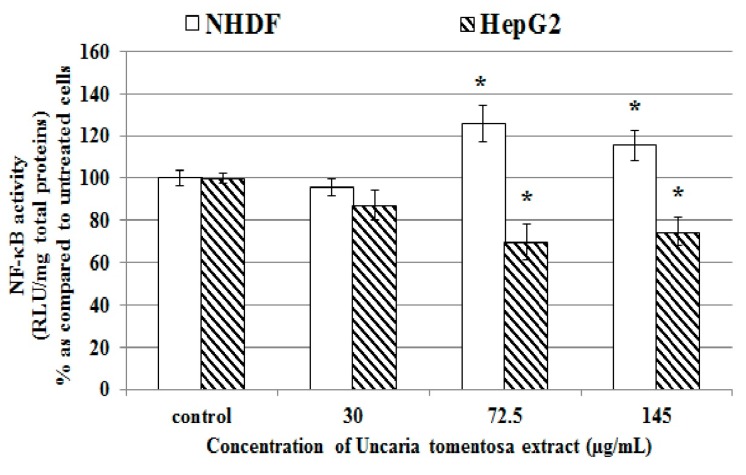
Changes in NF-κB activity in HepG2 and NHDF cells treated 4 h at 37 °C with *Uncaria tomentosa* leaves extract (30–145 µg/mL) according to the manufacturer’s protocol. Data are expressed as means ± SD from three independent experiments performed in triplicate. Statistically significant differences: * *p* < 0.05 refer to the control (untreated cells).

Oxidation-reduction potential of a cell has a direct impact on NF-κB regulation, which is involved in various processes, including cell proliferation and death [24]. Inconsistent data on effects of the individual components of *Uncaria tomentosa* as well as different extracts on modulation of the activity of NF-κB were reported [12,24]. The present study showed that incubation of HepG2 cells with the water *Uncaria tomentosa* extract resulted in about 25%–30% reduction (* *p* < 0.05) of NF-κB activity (Figure 8), while in NHDF cells incubated with this extract about 16%–26% increase (* *p* < 0.05) in NF-κB activity was noted.

The importance of the lack of enhanced NF-κB modulation for effectiveness of cancer therapy was indicated before [24] and recently supported by the study on the influence of C-Med100^®^ a standardized *Uncaria tomentosa* bark formulation on NF-κB and apoptosis induction in neoplastic cells [25]. Anticancer drugs used in clinical practice, including these used for hepatoma, i.e., cisplatin, inter alia through NF-κB activation may stimulate resistance of cancer cells to chemotherapy [23]. Inhibition of NF-κB activation should enhance effectiveness of cancer treatment. Reports concerning the effect of *Uncaria tomentosa* extracts on human leukemia cells (THP-1) indicated inhibition of the classical NF-κB activation pathway [26]. Both oxindole alkaloids [27] and procyanidins [28] have been found to possess the ability to inhibit NF-κB activation.

### 2.7. Effect of Uncaria tomentosa on CDDP Cytotoxicity against Cancer Cells

**Figure 9:**
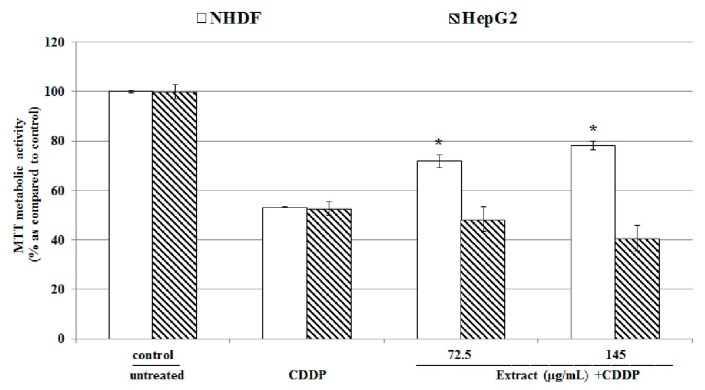
MTT assay (cell viability) results in NHDF and HepG2 treated 72 h at 37 °C with selected concentration of *Uncaria tomentosa* leaves extract (72.5–145 μg/mL) and the last 48 h also with CDDP (IC_50_ of 11.25 μM CDDP for HepG2 determined in our laboratory was used). Data are expressed as means ± SD from three independent experiments performed in triplicate. Statistically significant differences: * *p* < 0.05 refer to the CDDP alone treated cells.

The water *Uncaria tomentosa* extract decreased cancer cells viability (measured by MTT assay). Therefore, it can be hypothesized that it may enhance cytotoxic effect of anticancer drugs such as CDDP commonly used in clinical practice in cancer treatment. To test this hypothesis HepG2 and NHDF cells were 72 h incubated with tested extract and the last 48 h simultaneously with CDDP (at concentration corresponding to IC_50_ of 11.25 µM in HepG2 cells as recognized in our laboratory). Results of MTT assay (Figure 9) show that the water *Uncaria tomentosa* extract increased CDPP cytotoxic activity against HepG2 cells as indicated by 10%–15% decrease in cell viability.

However, when normal NHDF cells were treated with this extract (at concentrations of 72.5 and 145 µg/mL) and CDDP a significant (* *p* < 0.05) increase in cell viability by 30% as compared to NHDF cells treated only with CDDP was observed. This data suggests that tested *Uncaria tomentosa* decoction may enhance cytotoxic activity of CDDP against cancer cells and at the same time may protect normal cells against the harmful effects of cisplatin. Recently, it was reported that the use of *Uncaria tomentosa* capsules (300 mg/day) in patients with colorectal cancer. *Uncaria tomentosa* showed positive effects in reduced neutropenia and thrombocytopenia as well as in the repair of immune response [20]. Further studies are needed to confirm these observations. 

## 3. Materials and Methods

### 3.1. Plant Material and Decoction Preparation

Dried leaf blades of *Uncaria tomentosa* were acquired from controlled plantations of Laborations Induquinica (Lima, Peru), through Phytotherapy Center of Wilcaccora (Łomianki near, Warsaw, Poland). Comminuted dried leaf blades of *Uncaria tomentosa* (4 g) were extracted with warm water (200 mL, temperature below the boiling point) for 20 min. The decoction was subsequently centrifuged at 4000 rpm for 20 min. For cell culture experiments samples were separated by filtration. To analyze alkaloids and phenolic content the aqueous extract was lyophilized and as a result of extraction an average 1.16 g of dry powder was obtained.

### 3.2. HPLC—Analysis of Alkaloids

Determination of alkaloids in decoction of *Uncaria tomentosa* leaves was carried out according to Pilarski et al. [9]. To 100 mg of the dry powder (lyophilized decoction) 2% sulfuric acid solution (15 mL) was added and the mixture was sonicated for 15 min in an ultrasonic bath (Sonorex RK 103H, Bandelin, Berlin, Germany). The mixture was then centrifuged at 3000 rpm for 10 min and extracted tree times with 10 mL of ethyl acetate. The aqueous phase was separated and adjusted to pH 10 with 10% ammonium hydroxide, then extracted three times with 10 mL of ethyl acetate each. The organic extracts were combined, evaporated to dryness and the residue was dissolved in 10 mL of methanol. The qualitative and quantitative analysis of alkaloids was performed by HPLC using a LiChrospher 100 RP-18 (250 mm × 4 mm, Merck, Darmstadt, Germany) column and a LiChrospher 100 RP-18 (4 mm × 4 mm, Merck) precolumn; solvents: A—phosphate buffer solution (10 mM, pH 6.6), B—methanol:acetonitrile (1:1); gradient from (60% A and 40% B) to (30% A and 70% B); time: 30 min, flow rate 1 mL/min; detection at 245 nm with L-7450 Diode Array Detector (Merck-Hitachi, Darmstadt, Germany). The calibration curves were obtained from standard solutions of the alkaloids (isopteropodine, pteropodine, isomitraphylline, uncarine F, mitrophylline, speciophylline) in concentrations between 0.01 and 1.0 mg/mL. The standards of oxindole alkaloids were purchased from ChromaDex (Santa Ana, CA, USA). All standards came with NMR, MS and HPLC data. As an internal standard caffeine was used. Linear relationships between peak area and alkaloid concentration was observed. The results of the quantitative determination of alkaloids in the extract were presented in mg/100 g of extract dry weight.

### 3.3. Total Phenolic Content

The total phenolic content (TPC) was conducted on 96-well transparent microplates (Nunclon, Nunc, Roskilde, Denmark) according to the method previously described [29], using the Folin-Ciocalteu phenol reagent. Absorbance was measured at 680 nm after 20 min incubation with the Elisa Infinite Pro 200F reader (Tecan Group Ltd., Männedorf, Switzerland). TPC was determined using a standard curve prepared for gallic acid. Results were expressed as mg of gallic acid (GA) per 1 g of dry extract.

### 3.4. Total Flavonoid Content

Determination of total flavonoids was performed according to the method described by Lamaison and Carret [30] with some modifications [31]. Briefly, 20 µL of prepared sample was mixed on the microplate with 80 µL of methanol, 20 µL of 2% (*v*/*v*) AlCl_3_ methanol solution and filled with methanol to the volume of 200 µL. Absorbance was read at 430 nm after 30 min incubation with the solution containing methanol instead of the sample as a blank. Measurements were made using the Infinite Pro 200F microplate reader (Tecan Group Ltd.). Results are expressed as mg quercetin (Q) per 1 g of dry extract.

### 3.5. Tannin Content

To determine the content of tannins in our water extract (decoction), two methods were used: the vanillin assay and the protein precipitation method (precipitation of tannins with hide-powder).

### 3.6. Tannin Content Determined by Protein Precipitation Method

Analysis of tannin content was determined according to the hide-powder method described in the Polish Pharmacopoeia 6th edition [32]. Tannins were estimated indirectly after adsorption on and precipitation with insoluble hide powder. Result was expressed in milligram of pyrogallol (PGA) per gram of dry extract.

### 3.7. Tannin Content Determined by Vanillin/HCl Method

The amount of condensed tannins was determined using vanillin assay [33]. Absorbance was measured at 500 nm after 20 min incubation. The results were expressed as mg catechin (Cat) per 1 g of dry extract.

### 3.8. Cell Cultures

Human hepatoma (HepG2) were bought from American Type Culture Collection (Rockville, MD, USA) and cultured according to previously described procedures [22,34,35]. Experiments were conducted in DMEM with 10% FBS. Every 24 h the medium was changed for a fresh one with or without investigated concentrations of the extract. Human skin fibroblast cells (NHDFs) were purchased from Lonza (Walkersville, MD USA). NHDFs, cells (from the third to sixth passage) were seeded on 24-well plates (1.5–2.0 × 10^4^ cells per well) in FGM-2 with supplements and 2% FBS.

#### 3.8.1. Cell Viability Assessment (MTT Assay)

The cell viability was assessed by determination of MTT salt (3-(4,5-dimethylthiazol-2-yl)-2,5-diphenyltetrazolium bromide, Sigma-Aldrich Chemie, Darmstadt, Germany) conversion by mitochondrial dehydrogenase according to earlier described procedure [22]. Briefly, the cells were incubated for 72 h in 24-well plates with various concentrations of the extract, and subsequently for another 2 h with 0.5 mg/mL of MTT solution which is converted in live cells under the effect of mitochondrial dehydrogenase into insoluble formazan. The converted dye was then solubilized in 0.04 M HCl in absolute isopropanol. Absorbance of solubilized formazan was measured spectrophotometrically at 570 nm (BioTek Epoch microplate reader, BioTek Instruments Inc., Winooski, VT, USA). Cell viability was calculated as a percent versus the control (cells incubated in serum-free DMEM without extracts). The relative cell viability (%) was calculated as [A]/[B] × 100, where [A] is the absorbance of the test sample and [B] is the absorbance of control sample containing the untreated cells.

#### 3.8.2. Microscopic Examination

Microscopic examination of changes in cell morphology was assessed in visible light as described previously [22,35,36]. The assessment of viable and dead cells in fluorescence was performed after staining with calcein-AM and propidium iodide (PI), according to the protocol by MoBiTec (Gottingen, Germany). The digital images of viable cells were visualized using the phase-contrast inverted microscopy Eclipse TS 100F (Nikon, Melville, NY, USA) equipped with the Nikon Digital Sight DS-U2 camera using NIS-Elements Nikon BR 2.30 software (Nikon).

#### 3.8.3. Annexin V-FITC/PI Apoptosis Assay

Apoptosis was assessed by the AnnexinV-FITC Kit (Becton Dickinson, San Diego, CA, USA). The cells were incubated with *Uncaria tomentosa* extract for 72 h in 12-well plates. After the exposure, the culture medium was removed and the cells were harvested by trypsinization with 0.05% Trypsin-EDTA, and centrifuged at 2000 rpm for 10 min. The analysis by flow cytometery was performed within 1 h by counting 5000 gated events per each sample. The data were further analyzed using CELL-Quest software on FACS Calibur flow cytometer and the Cell Quest software (BD Biosciences, San Jose, CA, USA). The results were presented as rates of early apoptotic (Annexin V-positive) and late apoptotic (Annexin V and PI-positive) cells.

#### 3.8.4. ROS Production Measurement

ROS production was measured by the assessment of DCFH_2_-DA conversion and oxidation to DCF as was described previously [35]. DCFH_2_-DA, easily penetrates cells, undergoing conversion by esterase to DCFH_2_, which in the presence of ROS becomes oxidized to highly fluorescent DCF. Cells cultured in 12-well plates in serum-free DMEM were rinsed with PBS and incubated for 1 h in DMEM with investigated concentrations of the extract, and subsequently for 2 h with 250 µM of DCFH_2_-DA (2′,7′-dichlorodihydrofluorescein diacetate) in HBSS (Hank’s balanced salt solution). Fluorescence measurement was started immediately after DCFH2-DA was added, using a microplate reader (Synergy 4, BioTek Inc.) equipped with Gen5 software (BioTech Instruments Inc., Winooski, VT, USA) at an excitation wavelength of 485 nm and emission wavelength of 530 nm, with readings every 15 min.

#### 3.8.5. GSH Level Measurement

Cells were incubated on 24-well plates in serum free DMEM with the tested extract at various concentrations) for 4 h. The GSH level was assessed using the protocol for the glutathione assay kit (Sigma-Aldrich Chemie, Darmstadt, Germany). Absorbance measurement was performed at 412 nm using a microplate reader (Synergy 4, BioTek Inc.). The results were expressed in relation to the quantity of protein in the sample using the manufacturer’s protocol Pierce BCA Protein Assay Kit (Pierce Biotechnology, Rockford, IL, USA).

#### 3.8.6. Caspase-3 and -7 Activity Detection

The determination of caspase activity was performed with the use of the luciferase enzyme according to the protocol for the Caspase-Glo 3/7 Assay Kit provided by the manufacturer (Promega GmbH, Mannheim, Germany). Luminescence measurement was performed in cell lysates prepared using cells incubating in 96-well plates for 4 h with the tested extract. Luminescence intensity is proportional to caspase activity.

#### 3.8.7. NF-κB Active form Measurement

Chemiluminescent measurement of NF-κB active form was performed according to the protocol for NF-κB p65 ELISA Kit (Enzo Life Sciences, Farmigdale, NY, USA) in cell lysates prepared using cells incubating for 4 h with the tested extract. The results are presented in relation to the protein levels in the samples, measured by the Bradford’s method.

#### 3.8.8. Statistical Analysis

The results are presented as experimental means and SD. Statistical significance of observed differences was assessed by one-way analysis of variance (ANOVA) with the Tukey’s post-hoc test (Statistica ver. 8, StatSoft, Kraków, Poland) and as significant *p* < 0.05 was accepted.

## 4. Conclusions

Plant extracts and plant derived substances, and their usefulness in patients under anticancer therapies are now attracting great attention. They have shown promising effects when combined with chemotherapy and may benefit patients with hepatocellular carcinoma [36].

*Uncaria tomentosa*, also known as “cat’s claw” and “uña de gato”, is a Peruvian plant of the Rubiaceae family, whose bark, roots and leaves are commonly used by the local population in South America due to their immunomodulating, anticancer and anti-inflammatory action. Previous reports of the effects of *Uncaria tomentosa* on cancer cells focus on studies of bark and root extracts (with a high content of alkaloids), while its leaves contain similar active compounds and appropriate material could be collected without significant damages of the plant. However, differences in the content of polyphenols between bark and leaves as well as between aqueous and alcoholic extracts have been reported [5]. The extract used in the present study was prepared according to commonly accepted procedure for decoction preparation. It is free of the tetracyclic alkaloids, while contains pentacyclic oxindole alkaloids and condensed tannins.

This extract has been found to be cytotoxic against cancer cells and to induce oxidative stress in cancer cells but not in normal cells. In cancer cells the extract activates effector caspase-3 and caspase-7, which execute apoptosis through cleavage of protein substrates that include mediators and regulators of apoptosis, structural proteins, as well as DNA repair and cell-cycle related proteins. In addition in cancer but not in normal cells a reduction in NF-κB active form has been observed. Inhibition of NF-κB activity may prevent a development of cancer cell resistance to chemotherapy. Studied *Uncaria tomentosa* decoction enhances CDDP cytotoxicity against HepG2 cells, while incubation of NHDF cells with this extract prevents their viability reduction due to CDDP. These results indicate that *Uncaria tomentosa* leaves decoction modulates differently cancer and normal cells oxidative metabolism and enhances CDDP cytotoxicity against cancer cells. Every compound or combination of different compounds which is able to be more effective against cancer cells than against non-hyperplastic cells, may provide new therapeutic opportunities. Further studies are needed to describe molecular mechanisms underlying the observed effects and demonstrate the potential usefulness of the studied extract in increasing the sensitivity of cancer cells for treatment and/or in the prevention of drug resistance development. In general, the active ingredients of phytopharmaceuticals as used in ethnomedicine are mostly unknown and the plant extract is considered as active ingredient. The plant extract is a mixture of active compounds and their joint action can mediate pharmaceutical activity. Nevertheless, the isolation of active compounds should be the next step in order to elucidate the phytochemical basis of the effects observed in the current study.

## Figures and Tables

**Table 1:** Total content of phenolic compounds (TPC), flavonoids (TFC), and condensed tannin (CT) in dry (water) extract from leaves of *Uncaria tomentosa* measured by colorimetric assays and expressed as mg gallic acid (GA/g of dry extract), mg quercetin (Q/g of dry extract), mg catechin (Cat/g of dry extract) and mg pyrogallol (PGA/g of dry extract), respectively. Data are means ± SD from three independent experiments.

Sample	Total Phenolic Componds TPC (mg GA/g of Dry Extract)	Total Flavonoids TFC (mg Q/g of Dry Extract)	Condensed Tannin CT	
			Vanillin/HCl Method (mg Cat/g of Dry Extract)	Protein Precipitation Method (mg PGA/g of Dry Extract)
Dry water extract from *Uncaria tomentosa* leaves	242.37 ± 0.36	2.26 ± 0.09	21.81 ± 0.41	110.0 ± 2.0

## References

[1] Keplinger K., Laus G., Wurm M., Dierich M.P., Teppner H. (1999). *Uncaria tomentosa* (Willd.) DC. Ethnomedicinal use and new pharmacological, toxicological and botanical results. J. Ethnopharmacol..

[2] Heitzman M.E., Neto C.C., Winiarz E., Vaisberg A.J., Hammond G.B. (2005). Ethnobotany, phytochemistry and pharmacology of *Uncaria* (Rubiaceae). Phytochemistry.

[3] Wang G.F., Shi L.P., Ren Y.D., Liu Q.F., Liu H.F., Zhang R.J., Li Z., Zhu F.H., He P.L., Tang W. (2009). Anti-hepatitis B virus activity of chlorogenic acid, quinic acid and caffeic acid in vivo and in vitro. Antivir. Res..

[4] Sheng Y., Akesson C., Holmgren K., Bryngelsson C., Giamapa V., Pero R.W. (2005). An active ingredient of Cat’s Claw water extracts. Identification and efficacy of quinic acid. J. Ethnopharmacol..

[5] Bors M., Bukowska B., Pilarski R., Gulewicz K., Oszmiański J., Michałowicz J., Koter-Michalak M. (2011). Protective activity of the *Uncaria tomentosa* extracts on human erythrocytes in oxidative stress induced by 2,4-dichlorophenol (2,4-DCP) and catechol. Food Chem. Toxicol..

[6] Sheng Y., Pero R.W., Amiri A., Bryngelsson C. (1998). Induction of apoptosis and inhibition of proliferation in human tumor cells treated with extracts of *Uncaria tomentosa*. Anticancer Res..

[7] Riva L., Coradini D., Di Fronzo G., De Feo V., De Tommasi N., De Simone F., Pizza C. (2001). The antiproliferative effects of *Uncaria tomentosa* extracts and fractions on the growth of breast cancer cell line. Anticancer Res..

[8] Cheng A.C., Jian C.B., Huang Y.T., Lai C.S., Hsu P.C., Pan M.H. (2007). Induction of apoptosis by *Uncaria tomentosa* through reactive oxygen species production, cytochrom c release, and caspases activation in human leukemia cells. Food. Chem. Toxicol..

[9] Pilarski R., Poczekaj-Kostrzewska M., Ciesiołka D., Szyfter K., Gulewicz K. (2007). Antiproliferative activity of various *Uncaria tomentosa* preparations on HL-60 promyelocytic leukemia cells. Pharmacol. Rep..

[10] Pilarski R., Filip B., Wietrzyk J., Kuraś M., Gulewicz K. (2010). Anticancer activity of the *Uncaria tomentosa* (Willd.) DC. Preparations with different oxindole alkaloid composition. Phytomedicine.

[11] Bacher N., Tiefenthaler M., Sturm S., Stuppner H., Ausserlechner M.J., Kofler R., Konwalinka G. (2006). Oxindole alkalois from *Uncaria tomentosa* induce apoptosis in proliferating, G0/G1-arrested and bcl-2-expressing acute lymphoblastic leukaemia cells. Br. J. Haematol..

[12] Prado E.G., Gimenez M.D.G., Vázquez R.P., Sánchez J.L.E., Rodríguez M.T.S. (2007). Antiproliferative effects of mitraphylline, a pentacyclic oxindole alkaloid of *Uncaria tomentosa* on human glioma and neuroblastoma cell lines. Phytomedicine.

[13] Rizzi R., Re F., Bianchi A., De Feo V., de Simone F., Bianchi L., Stivala L.A. (1993). Mutagenic and antimutagenic activities of *Uncaria tomentosa* and its extracts. J. Ethnopharmacol..

[14] Dreifuss A.A., Bastos-Pereira A.L., Fabossi I.A., Lívero F.A., Stolf A.M., Alves de Souza C.E., Gomes Lde O., Constantin R.P., Furman A.E., Strapasson R.L (2013). *Uncaria tomentosa* exerts extensive anti-neoplastic effects against the Walker-256 tumour by modulating oxidative stress and not by alkaloid activity. PLoS ONE.

[15] Forner A., Hessheimer A.J., Real M.I., Bruix J. (2006). Treatment of hepatocellular carcinoma. Crit. Rev. Oncol. Hematol..

[16] Jo K.J., Cha M.R., Lee M.R., Yoon M.Y., Park H.R. (2008). Methanolic extracts of Uncaria rhynchophylla induce cytotoxicity and apoptosis in HT-29 human colon carcinoma cells. Plant Foods Hum. Nutr..

[17] Wagner H., Kreutzkamp B., Jurcic K. (1985). Die Alkaloide von *Uncaria tomentosa* und ihre Phagozytose-steigernde Wirkung. Planta Med..

[18] García Giménez D., García Prado E., Sáenz Rodríguez T., Fernández Arche A., De la Puerta R. (2010). Cytotoxic effect of the pentacyclic oxindole alkaloid mitraphylline isolated from *Uncaria tomentosa* bark on human Ewing’s sarcoma and breast cancer cell lines. Planta Med..

[19] Wurm M., Kacani L., Laus G., Keplinger K., Dierich M.P. (1998). Pentacyclic oxindole alkaloids from *Uncaria tomentosa* induce human endothelial cells to release a lymphocyte-proliferation-regulating factor. Planta Med..

[20] Farias I.L., Araújo M.C., Farias J.G., Rossato L.V., Elsenbach L.I., Dalmora S.L., Flores N.M., Durigon M., Cruz I.B., Morsch V.M. (2012). *Uncaria tomentosa* for Reducing Side Effects Caused by Chemotherapy in CRC Patients: Clinical Trial. Evid. Based Complement. Alternat. Med..

[21] Khanbabaee K., van Ree T. (2001). Tannins: Classification and definition. Nat. Prod. Rep..

[22] Grudzinski I.P., Bystrzejewski M., Cywinska M.A., Kosmider A., Poplawska M., Cieszanowski A., Fijalek Z., Ostrowska A., Parzonko A. (2014). Assessing carbon-encapsulated iron nanoparticles cytotoxicity in Lewis lung carcinoma cells. J. Appl. Toxicol..

[23] Reuter S., Gupta S.C., Chaturvedi M.M., Aggarwal B. (2010). Oxidative stress, inflammation, and cancer, How are they linked?. Free Radic. Biol. Med..

[24] Sandoval M., Charbonnet R.M., Okuhama N.N., Roberts J., Krenova Z., Trentacosti A.M., Miller M.J. (2000). Cat’s claw inhibits TNFalpha production and scavenges free radicals: Role in cytoprotection. Free Radic. Biol. Med..

[25] Akesson C., Lindgren H., Pero R.W., Leanderson T., Ivars F. (2005). Quinic acid is a biologically active component of the *Uncaria tomentosa* extract C-Med 100^®^. Int. Immunopharmacol..

[26] Allen-Hall L., Arnason J.T., Cano P., Lafrenie R.M. (2010). *Uncaria tomentosa* acts as a potent TNF-α inhibitor through NF-κB. J. Ethnopharmacol..

[27] Valerio L.G., Gonzales G.F. (2005). Toxicological aspects of the South American herbs cat’s claw (*Uncaria tomentosa*) and Maca (*Lepidium meyenii*), a critical synopsis. Toxicol. Rev..

[28] Terra X., Valls J., Vitrac X., Mérrillon J.M., Arola L., Ardèvol A., Bladé C., Fernandez-Larrea J., Pujadas G., Salvadó J. (2007). Grape-seed procyanidins act as antiinflammatory agents in endotoxin-stimulated RAW 264.7 macrophages by inhibiting NF-κB signaling pathway. J. Agric. Food Chem..

[29] Olech M., Nowak R., Los R., Rzymowska J., Malm A., Chruściel K. (2012). Biological activity and composition of teas and tinctures prepared from *Rosa rugosa* Thunb. Cent. Eur. J. Biol..

[30] Lamaison J.L.C., Carret A. (1990). Teneurs en principaux flavonoids des fleurs de *Crataegus monogyna* Jacq et de *Crataegus laevigata* (Piret DC) en fonction de la vegetation. Plantes Méd. Phytothér..

[31] Pietrzak W., Nowak R., Olech M. (2014). Effect of extraction method on phenolic content and antioxidant activity of mistletoe extracts from *Viscum album* subsp. Abietis. Chem. Pap..

[32] (2005). Polish Pharmacopoeia.

[33] Price N.J., van Scoyoc S., Butler L.G. (1978). A critical evaluation of the vanillic reactions in an assay for tannin in sorghum grain. J. Agric. Food Chem..

[34] Jaszewska E., Kośmider A., Kiss A.K., Naruszewicz M. (2009). Pro-oxidative and pro-apoptotic action of defatted seeds of *Oenothera paradoxa* on human skin melanoma cells. J. Agric. Food Chem..

[35] Grudzinski I.P., Bystrzejewski M., Cywinska M.A., Kosmider A., Poplawska M., Cieszanowski A., Fijalek Z., Ostrowska A. (2014). Comparative cytotoxicity studies of carbon-encapsulated iron nanoparticles in murine glioma cells. Colloids Surf. B Biointerfaces.

[36] Shu X., McCulloch M., Xiao H., Broffman M., Gao J. (2005). Chinese herbal medicine and chemotherapy in the treatment of hepatocellular carcinoma: A meta-analysis of randomized controlled trials. Integr. Cancer Ther..

